# Toxicological assessment of polyhexamethylene biguanide for water treatment

**DOI:** 10.1515/intox-2015-0029

**Published:** 2015-12

**Authors:** Isaac J. Asiedu-Gyekye, Abdulai Seidu Mahmood, Charles Awortwe, Alexander K. Nyarko

**Affiliations:** 1 Department of Pharmacology and Toxicology, University of Ghana School of Pharmacy, College of Health Sciences, Legon, Ghana; 2 Department of Pathology, School of Biomedical and Allied Health Sciences, College of Health Sciences, Korle-Bu, Ghana; 3 Division of Pharmacology, Faculty of Health Sciences, University of Stellenbosch, Cape Town, South Africa

**Keywords:** polyhexamethylene biguanide, toxicity, biochemical hematology, histopathology, LD_50_, therapeutic index

## Abstract

Polyhexamethylene biguanide (PHMB) is an antiseptic with antiviral and antibacterial properties used in a variety of products including wound care dressings, contact lens cleaning solutions, perioperative cleansing products, and swimming pool cleaners. There are regulatory concerns with regard to its safety in humans for water treatment. We decided to assess the safety of this chemical in Sprague-Dawley rats. PHMB was administered in a single dose by gavage via a stomach tube as per the manufacturer's instruction within a dose range of 2 mg/kg to 40 mg/kg. Subchronic toxicity studies were also conducted at doses of 2 mg/kg, 8 mg/kg and 32 mg/kg body weight and hematological, biochemical and histopathological findings of the major organs were assessed. Administration of a dose of 25.6 mg/kg, *i.e.* 1.6 mL of 0.4% PHMB solution (equivalent to 6.4x10^3^ mg/L of 0.1% solution) resulted in 50% mortality. Histopathological analysis in the acute toxicity studies showed that no histopathological lesions were observed in the heart and kidney samples but 30% of the animals had mild hydropic changes in zone 1 of their liver samples, while at a dosage of 32 mg/kg in the subchronic toxicity studies, 50% of the animals showed either mild hepatocyte cytolysis with or without lymphocyte infiltration and feathery degeneration. Lymphocyte infiltration was, for the first time, observed in one heart sample, whereas one kidney sample showed mild tubular damage. The acute studies showed that the median lethal dose (LD_50_) is 25.6 mg/kg (LC_50_ of 1.6 mL of 0.4% PHMB. Subchronic toxicological studies also revealed few deleterious effects on the internal organs examined, as seen from the results of the biochemical parameters evaluated. These results have implications for the use of PHMB to make water potable.

## Introduction

Polyhexamethylene biguanide (PHMB) is an antiseptic with antiviral and antibacterial properties used in several ways including wound care dressings, contact lens cleaning solutions, perioperative cleansing products, and swimming pool cleaners. It is also known as *polyhexanide and polyaminopropyl biguanide, polymeric biguanide hydrochloride; polyhexanide biguanide.* It is a commonly applied antiseptic, often used as a preservative in cosmetics and personal care products (Schnuch *et al*., 2007).

The antimicrobial efficacy has been demonstrated on *Acanthamoeba polyphaga, A castellanii,* and *A hatchetti* (Hughes *et al*., 2003; Wright *et al*., 2003; Burgers *et al*., 1994; Hiti *et al*., 2002). *In vivo* studies have also demonstrated that a miltefosine–polyhexamethylene biguanide combination is highly effective for the treatment of Acanthamoeba keratitis (Polat *et al*., 2013).

As a biocide, additional pharmacological effects have been demonstrated against *Legionella pneumophila,* against gram positive and gram negative bacteria. It is a broad spectrum virucide and has amebicidal activities (Gilbert *et al*., 1990; Kramer *et al*., 2004; Broxton *et al*., 1984; Lee *et al*., 2007). PHMB retains its activity in hard water and does not cause surface streaks or tackiness (Broxton *et al*., 1984b; Ikeda *et al*., 1984). Consistent with previous studies, a PHMB mouthrinse was shown to inhibit plaque re-growth and reduced oral bacterial counts, indicating that PHMB could be an alternative to established mouth rinses in preventive applications (Welk *et al*., 2005). Recreational water maintained and sanitized with PHMB is however assumed to serve as a medium for transmission of ocular adenovirus infections, mainly because at a concentration of 50 ppm, PHMB was not virucidal against adenovirus at temperatures consistent with swimming pools or hot tubs (Romanowski *et al*., 2013).

Previous studies have shown increased frequency of sensitization to 0.5% and 0.4% PHMB in unselected dermatitis patients (Schnuch *et al*., 2007). PHMB proved also toxic to keratocytes (Lee *et al*., 2007) and was shown to have acute toxic effects in human cells where it caused severe inflammation, atherogenesis, and aging. Moreover, PHMB produced embryo toxicity and heart failure in zebrafish (Kim *et al*., 2013).

Though, officially not used in the treatment of drinking water, there have been instances where toxic effects were experienced in certain individuals. For example in the period from August 2006 to May 2007, more than 12,500 patients were admitted to hospital with a history of drinking illegal cheap “vodka” in 44 different regions in Russia, of whom 9.4% died. In reality, the “vodka” was an antiseptic liquid composed of ethanol (≈93%), diethyl phthalate, and 0.1-0.14% polyhexamethylene guanidine (PHMG) (“Extrasept-1”) (Ostapenko *et al*., 2011). Previous studies have also shown that another biocide – polyhexamethylene guanidine hydrochloride – has an LD_50_ of 600 mg/kg in rats (Asiedu-Gyekye *et al*., 2014). There have been various regulatory concerns with regard to the use of these biocides in water treatment. We therefore evaluated the safety of PHMB when used in treating water to make it potable and also in the case of survivors of drowning events, concentrating especially on its effect on the major organs.

## Materials and methods

PHMB concentrate was purchased from AGRIMAT-Ghana as an aqueous solution. A stock solution of 0.1% concentration of PHMB was prepared using deionized water. This was equivalent to 1.0 mg/mL of PHMB. Further dilutions were made using deionized water.

### Animal husbandry and groupings

Eight-week-old Sprague-Dawley rats (250 g body weight) of both sexes were acquired from Noguchi Memorial Institute for Medical Research, University of Ghana, Legon and housed in rooms with regulated room temperature of 26°C and humidity of 40 to 60%. The animals were exposed to 12 h light and 12 h darkness. The females were nulliparous and non-pregnant. The animals were randomly assigned to 4 groups of 10 animals each for the acute toxicity test. A similar provision was made for the subchronic study. Animal feed (Kosher Feed Mills Ltd, Osu, Accra) and water were given *ad libitum.* To ensure effective absorption from the gastrointestinal tract after oral administration, feed was withdrawn 8 h prior to treatment and further withheld for an extra 30 min after administration of PHMB before being reintroduced. Equal numbers of rats were randomized and each marked in their individual cages for 7 days prior to PHMB administration. Equal numbers of animals of both sexes were used at each dose level of PHMB.

### Acute toxicity

PHMB was administered as a single dose by gavage in view of the potential mode of ingestion. The animals received doses of 2 mg/kg (500 mg/L), 4 mg/kg (2000 mg/L), 32 mg/kg (8000 mg/L) and 40 mg/kg (10000 mg/L of 0.1% PHMB solution). Since the maximum volume of liquid that could be administered was 1 mL/100 g of body weight, an appropriate adjustment was made in preparing the concentrations so as to avoid exceeding the recommended volume of not more than 2 mL for oral administration (Lee, 1985). Thus 5 different concentrations were prepared. Control animals received only deionized water. The animals were observed every 30 min for the first 4 h, and every 8 h for the next 24 h. The number of animals that died within the 24 h period was recorded for each treatment. The rest of the animals were observed daily for 14 days and any clinical signs were recorded. Clinical signs monitored included respiratory distress, frequency of urination, swellings, abnormal gait, *etc.*

At the end of the study, the animals were euthanized and blood samples drawn from the descending aorta for hematological and clinical chemistry. Hematological analysis was done using SysMex K-21 (Sysmex Corporation, Japan, 2003), whereas clinical chemistry was done using Microlab 300 equipment (Sysmex Corporation, 2003). Necropsy was performed on the animals and samples of kidneys, heart and liver were harvested and immediately fixed in 10% buffered formalin. The samples were dehydrated in graded alcohols, cleared in xylene and embedded in paraffin wax. Multiple sections of 4 μ m were cut using a microtone and stained with hematoxylin and eosin (H&E).

### Sub-chronic toxicity

Eight week-old Sprague-Dawley rats of mean weight 250 g of both sexes were randomly assigned to 4 groups of 6 animals each. They were housed and treated as described above. Three dosage levels were continuously administered to the rats for a period of 60 days. The doses administered were as follows:

♦ **Group 1:** 2 mg/kg (equivalent to 0.2 mg/L of 0.4% solution of PHMB)
♦ **Group 2:** 8 mg/kg (equivalent to 0.4 mg/L of 0.4% solution of PHMB)
♦ **Group 3:** 32 mg/kg (equivalent to 1.2 mg/L of 0.4% solution of PHMB)
♦ **Control:** deionized water

Following administration, the animals were initially observed every hour for the first 6 hours and subsequently every 6 hours for 24 hours, then daily for 14 days. The animals were monitored for clinical signs including respiratory distress, frequency of urination, swellings, gait, *etc.* They were also weighed every 3 days to monitor any weight changes. Eventually the animals were sacrificed and kidneys, livers and hearts were harvested for histopathological studies. Blood samples were taken and analyzed as described earlier. All experimental procedures were conducted in accordance with the internationally acceptable guidelines for evaluating the safety and efficacy of herbal medicines (WHO, 2000 and OECD, 2001).

### Statistical analysis

Statistics was performed using Graphpad Prism 5. Means ± SEM were determined for quantitative variables. Analysis of variance (ANOVA) was used to determine statistical significance invariables among the groups at p-values ≤0.05 followed by Bonferroni *post hoc* analysis.

## Results

### Acute toxicity

Preliminary studies showed that administration of a dose of 20 mg/L to rats did not result in death of any animal. However death resulted few minutes after administration of a dose as high as 1.6 mL of 0.4% PHMB solution, which was equivalent to a dose of 6.4×10^3^ mg/L of 0.1% solution. Mortality results from the present study are shown in Table 1.

**Table 1:** Mortality rate of rats orally administered various concentrations of PHMB solution.

Treatment group	Number of animals		Number of deaths and clinical manifestations	% Dead
	Start	End		
Control - Deionized water	10	10	NIL (normal behavior)	0%
2 mg/kg (500 mg/L of 0.1%) PHMB	10	10	NIL (normal movements and 4 with reduced response to stimuli)	0%
8 mg/kg (2000 mg/L of 0.1%) PHMB	10	8	2 (after 6 hours tonic-clonic movements with repetitive circling)	20%
25.6 mg/kg (6400 mg/L of 0.1%) PHMB	10	5	5 (2 within 5 minutes, 1 after 1 hour and 2 within 24hours with tonic-clonic convulsions)	50%
32 mg/kg (8000 mg/L of 0.1%) PHMB	10	2	8 (6 within 6 hours and 2 within 24 hours)	80%
40 mg/kg (10000 mg/L of 0.1%) PHMB	10	NIL	10 (all within 1 hour with convulsions, changes in gait and respiratory distress)	100%

### Clinical manifestations

At the dose that produced the LD_50_, the animals presented with arched-back posture and partially closed eyes, inactivity, impaired response to sound and sight, ataxia, diarrhea, hyperreflexia, and convulsive twitching.

In the subchronic studies, immediately after dosing, hunched posture and pilo-erection were noted in 5 animals treated with 32 mg/kg. Signs of systemic toxicity were also noted 2 days after dosing in 1 animal treated with 32 mg/kg, exhibiting lethargy, ataxia, decreased respiratory rate, labored respiration, ptosis and tiptoe gait. All the animals appeared healthy with no apparent change in feeding habits during and after treatment. With regard to clinical manifestations, no debilitating clinical sign was evident, and neither were there any significant changes in weight of the animals during the period.

No death was recorded in any of the treatment groups during the observation period. There were little or no changes in body weight of the animals over the experimental period.

### Hematological parameters

**Figure 1:**
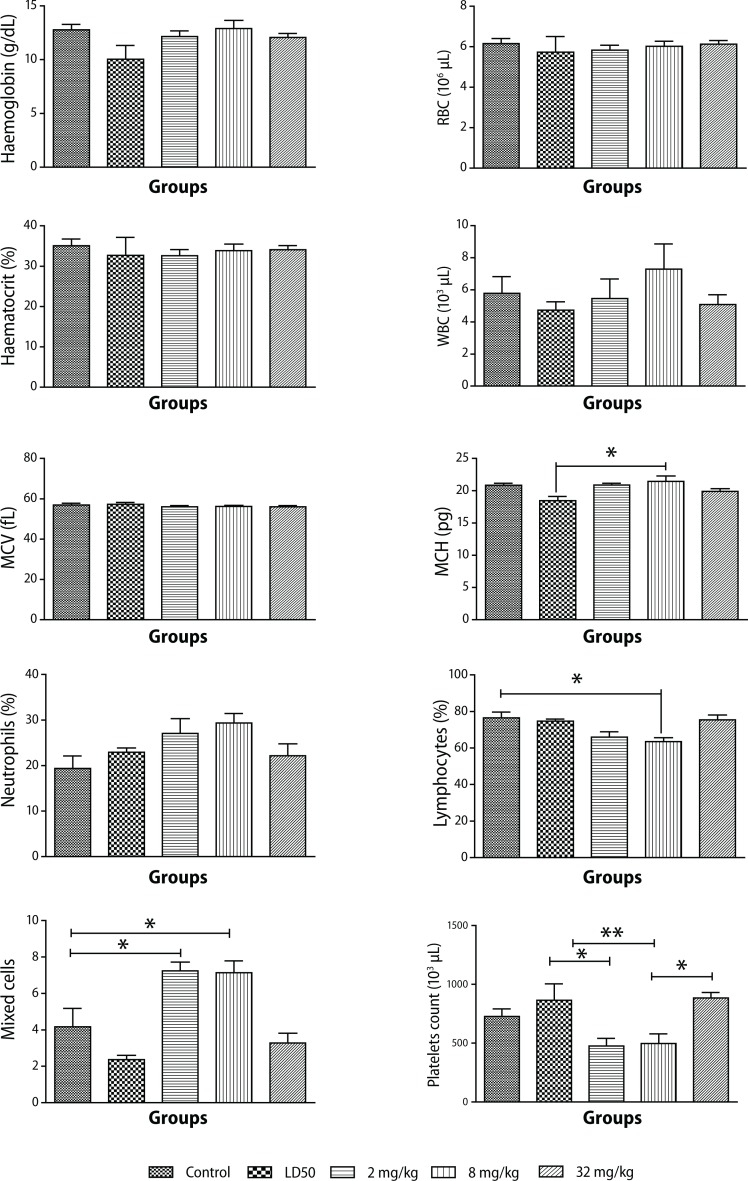
Changes in hematological parameters during PHMB administration in Sprague-Dawley rats. Values are expressed as mean ± SEM, n=10. The differences among the means were analyzed using two-way ANOVA and values considered significant when *p*<0.05. No significant changes were observed in hematocrit, RBC, and MCV levels. There was an increase in WBC count (*p*<0.55) and a slight increase in MCH (*p*<0.012) compared to animals that received the LD_50_ PHMB. There was a reduction in lymphocyte count (*p*<0.0055) as compared to control in animals that received 8 mg/kg PHMB. There was also a reduction in platelet count (*p*<0.0014) compared to the LD_50_ group. An increase in neutrophil levels (*p*<0.103) as compared to controls was also observed. Mixed cell levels significantly increased (*p*<0.0001) in animals that received 2 mg/kg and 8 mg/kg PHMB as compared to controls.

In the acute studies, there were no significant differences in the RBC counts between the control and the treated animals (Figure 1a).

The mean hemoglobin concentration was reduced in animals undergoing acute toxicity studies at the LD_50_ level compared to controls.

Animals treated subchronically showed no changes at any of the doses tested compared to the control group. Mean Corpuscular Hemoglobin (MCH) remained on the average unchanged regardless of the dose administered, and so did mean Corpuscular Hemoglobin Concentration (MCHC) (Figure 1a).

Compared with the control, a slight decrease in white blood cell (WBC) count was observed in all the doses given, except the recommended one. Nevertheless, there were no significant differences between values at *p*<0.05.

A slight decrease in leukocyte count was evident in samples from rats treated with 2 mg/kg and 8 mg/kg of PHMB, but no such observation was made in rats in the other groups compared to controls (Figure 1a).

High levels of mean neutrophil count were observed at doses of 2 mg/kg and 8 mg/kg. Higher doses, including the dose for LD_50_, yielded lower values (Figure 1b).

Platelet count was reduced by almost 30% at the lowest dose compared to controls. Platelets in the rats treated with the highest dose in the subchronic and in the acute toxicity studies, on the other hand, were relatively higher than in controls (Figure 1b).

### Biochemical parameters

#### Liver function tests (LFTs)

**Figure 2:**
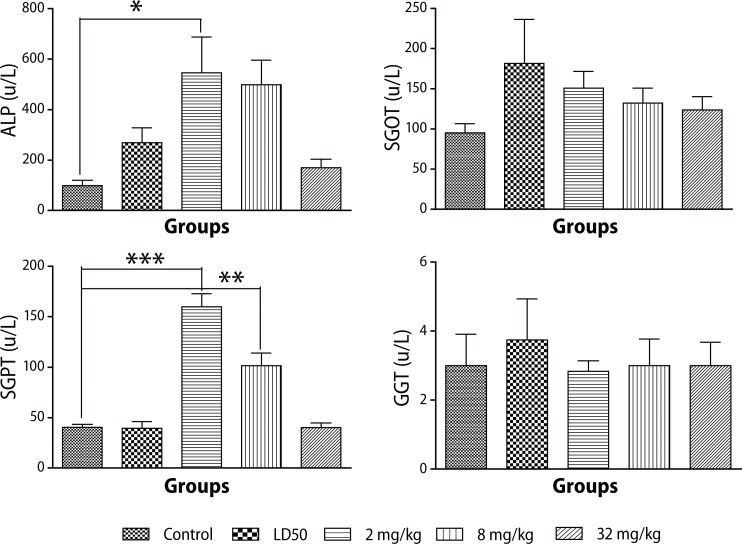
Changes in liver enzymes during administration of different doses of PHMGH in Sprague-Dawley rats. Values are expressed as mean ± SEM, n=10. The differences among the means were analyzed using two-way ANOVA and values considered significant when *p*<0.05. There was a significant increase in ALP levels (*p*<0.0086) observed in animals that received 2 mg/kg PHMB and an increase in SGPT (ALT) levels (*p*<0.0001) in animals that received 2 mg/kg and 8 mg/kg.

High levels of alkaline phosphatase (ALP) were observed at doses of 2 mg/kg and 8 mg/kg, but ALP levels for the dose of 32 mg/kg were comparable to the control level. High levels of serum glutamic oxaloacetic transaminase (SGOT or AST) U/L and SGPT or ALT (*p*<0.0001) were also observed in the acute toxicity study. However levels of both SGOT and SGPT decreased with increasing doses of PHMB solution administered. Besides, their mean levels obtained for controls were even lower than for the treated groups (Figure 2).

High levels of gamma glutamyl transpeptidase (GGT) were observed at the dose that produced the LD_50_ value, but no significant change was observed during the subchronic study (Figure 2).

#### Lipid profile

**Figure 3:**
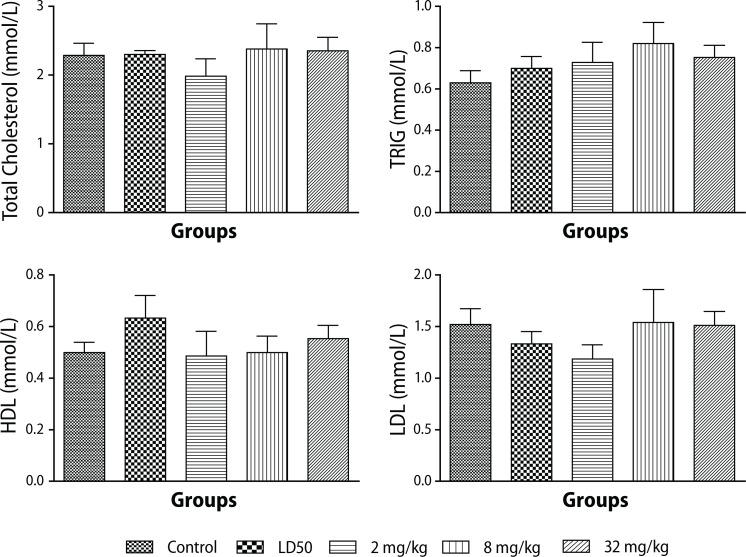
Changes in lipid profile during administration of different doses of PHMB in Sprague-Dawley rats. Values are expressed as mean ± SEM, n=10. The differences among the means were analyzed using two-way ANOVA. The differences among the mean values were considered significant when *p*<0.05. There were no significant changes in the lipid profile. A slight increase in TRIG levels (*p*<0.6325) as compared to controls.

None of the lipid parameters measured showed any significant differences compared to the control groups (Figure 3).

#### Renal function tests

**Figure 4:**
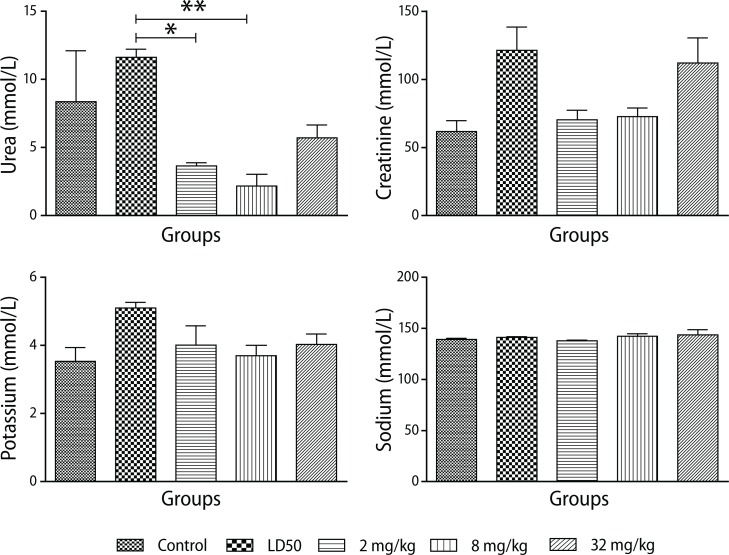
Changes in renal profile during administration of different doses of PHMB in Sprague - Dawley rats. Values are expressed as mean ± SEM, n=10. The differences among the means were analyzed using two-way ANOVA and considered significant when *p*<0.05. A significant reduction in urea levels (*p*<0.0025) as compared to animals that received deionized water (control group) and LD_50_ group. Creatinine levels increased (*p*<0.013) in animals that received 32 mg/kg PHMB compared to controls.

A very high concentration of urea was observed in the acute toxicity studies. The subchronic studies revealed low urea levels at the lowest dose of 2 mg/kg (*p*<0.0025) with a slight increase at the highest dose of 32 mg/kg. The control value was also found to be relatively high but was not significantly different from the treated groups (Figure 4). Creatinine levels in the test and control groups were comparable (*p*<0.013).

#### Electrolyte profile

**Figure 5:**
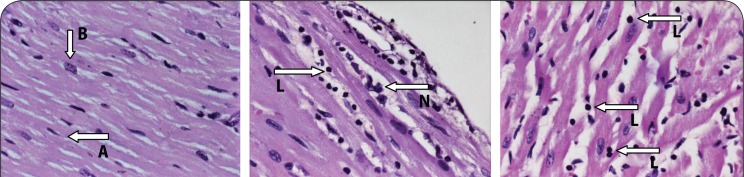
Representation of a section of the heart muscle from a rat showing a) normal myocardial fibers in control animals that received deionized water only in the acute toxicity group (note the characteristic normal branching (A) and central placed nuclei (B) of myocardial fibers), b) mild myocarditis in rats that received 32 mg/kg PHMB for 90 days (note the presence of few leukocyte infiltrates: lymphocyte (L) and neutrophil (N) and the apparent absence of myocardial degeneration), H&E stain; ×40.

A relativeely high mean potassium concentration was observed in acute toxicity studies but the levels between the treatment groups and the controls were not statistically different. Similar observations were made for sodium concentrations in the control and test groups (Figure 5b).

### Pathological analysis GROSS PATHOLOGY

#### Acute toxicity

The acute toxicity showed evidence of gastroenteritis, congestion of the lungs, liver and kidneys, yet all the organs harvested from subchronic studies were comparable with the controls in appearance, size and consistency.

#### Subchronic toxicity

There were no visible pathological changes in the heart, kidney and liver samples when different dose levels of PHMB were administered to the animals.

### HISTOPATHOLOGICAL STUDIES

#### Acute toxicity

**Figure 7:**
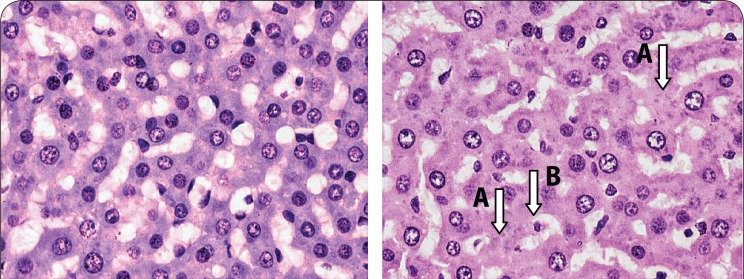
Hematoxylin and eosin stained section of a liver from a rat, a) from the control group that received only deionized water showing normal hepatocytes, b) from a rat that received the LD_50_ dose of PHMB, 8 mg/kg and 32 mg/kg PHMB showing mild hepatocellular necrosis. Note the presence of anucleated cells (A) and cells with pyknotic nucleus (B) indicative of mild hepatic injury; ×40.

No histopathological lesions were observed in the heart and kidney samples, but 30% of the animals had mild hydropic changes in zone 1 of their liver samples (Figure 7b).

#### Subchronic toxicity

**Figure 6:**
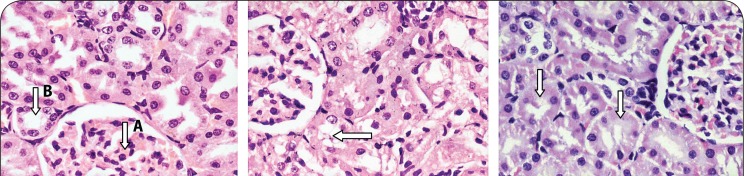
a) Section from the kidney of a rat showing a) normal morphology of a glomerulus (A) and tubules (B) in controls that received only deionized water, b) mild tubular damage during sub- chronic toxicity studies (2 mg/kg, 8 mg/kg, and 32 mg/kg) showing the presence of anucleated tubular epithelial cells and mild disruption of tubular architecture (arrow), c) severe tubular damage during subchronic toxicity studies (32 mg/kg) revealing the presence of anucleated tubular epithelial cells and disruption of tubular architecture (arrows). H&E stain; ×40.

None of the heart and liver samples from control animals had any histopathological lesions (Figure 5a and 7a). However, the kidneys of 30% of the animals showed mild tubular damage (Figure 6b), whereas severe necrotic tubular damage was seen only in one sample (Figure 6c).

At the dose of 2 mg/kg there were no lesions observed in any of the 6 samples of liver or heart investigated. However, 50% of the kidney samples showed evidence of mild tubular damage (Figure 6b). At the test concentration of 8 mg/kg, the heart samples showed no lesions, but 50% of liver samples showed evidence of either mild hepatocyte cytolysis or feathery degeneration with or without increased lymphocyte infiltration. Fifty percent of kidney samples were found to have developed mild tubular necrosis.

One rat treated with the highest dose of 32 mg/kg showed mild tubular damage, while 50% of liver samples tested at the same dose showed either mild hepatocyte cytolysis with or without lymphocyte infiltration and feathery degeneration. Lymphocyte infiltration was, for the first time, observed in one heart sample (Figures 5b and 5c) and one kidney sample also showed mild tubular damage.

#### Histopathology

The hematoxylin and eosin stained sections of the liver, heart and kidney samples of the sacrificed animals were examined by a qualified pathologist at the College of Health Sciences.

Normal liver sections showed the characteristic hexagonal arrangement of hepatocytes in lobules surrounding a central vein. Sections from a normal heart showed characteristic branching arrangement of myocardial fibers with centrally placed nuclei.

Normal kidney sections revealed well organized sections through the tubules and numerous glomeruli. No disturbance in the architecture of these organs was considered pathological.

Acute PHMB-mediated disturbances in the liver could constitute fluid accumulation from exudates, infiltration of normal cellularity with leukocytes and some disturbances in normal hepatocytes, such as degeneration of individual hepatocytes by loss of nuclear (karyorrhexis) or pyknosis. Myocarditis in this study was characterized by myocardial inflammation (inflammation of the muscular layer) resulting in injury to the cardiac myocytes and infiltration of the muscle by leukocytes (lymphocytes and neutrophils). Kidney disturbances were in the form of tubular or glomerular degeneration, in the form of structural disruption and nuclear chromatin condensation (pyknosis) or complete nuclear loss (karyohexis).

## Discussion

This study aimed to assess the safety level of PHMB when used to sanitize water to make it potable. The LD_50_ calculated from the study was found to be 25.6 mg/kg (equivalent to 6.4x10^3^ mg/L of 0.1% PHMB solution). Blood chemistry studies also indicated little or no adverse reaction on cellular components of the blood. All the indices examined were comparable to those of controls, suggesting that the chemical may not have any adverse effects on cellular components of the blood at doses below 25.6 mg/kg, the dose which elicited LD_50_.

Potassium concentrations detected were very high compared to controls, *p*<0.0057, suggesting that at LD_50_ level most of the rats might have experienced abnormal heart beats, but this was not confirmed by the histopathological study on the heart as there were no cellular lesions detected. This finding may suggest that in spite of the high dose tested, which possibly might have caused abnormal heart beat in some animals, the integrity of the architecture of the vital organs was not compromised. This observation is similar to that made for PHMGH (Asiedu-Gyekye *et al*., 2014). Sodium concentrations, on the other hand, did not show any change in either

PHMB-treated or control groups (*p*<0.08), thus most of the clinical manifestations such as lethargy, weakness, etc, usually associated with sodium imbalance were not observed in the study. It is worth noting that most of the animals that died exhibited various nervous manifestations such as abnormal gait and tonic-clonic convulsions. These observations were not supported by the electrolyte profile obtained from blood chemistry analysis. A chronic toxicity study, which is beyond the goal of this study, is recommended to be carried out to further investigate this nervous phenomenon. It should be emphasized that mortality only occurred at very high doses.

Blood biochemistry included analysis of AST, ALT, GGT and lipid profile. ALT and AST are found usually in the liver, but small quantities are found in kidneys, muscles and pancreas (Friedman & Keefe, 2012). The ALT and AST levels in PHMB-treated rats were comparable to those of controls, suggesting that the integrity of the liver cell membrane was not compromised by PHMB treatment. The histopathological study, however, revealed mild hepatic injuries in 50% of animals at a high dose of treatment, suggesting that the integrity of the liver, and perhaps of all the other organs, may have been compromised. Biochemical analysis, carried out for all the animals, indicated that there was no significant difference between the control and the treated groups, lending further support to the assertion that the chemical may not be toxic to rats at the manufacturer's recommended dose of 8 mg/kg. Acute liver injury has however been associated with the use of another biocide, polyhexamethylene guanidine hydrochloride (PHMGH 0.1–0.14%) or PHMG ingested with either ethanol or diethyl phthalate. The injury caused following such ingestion produced lesions similar to cholestatic hepatitis with a severe inflammatory component causing high mortality (Ostapenko *et al*., 2011).

In the acute toxicity study, high levels of urea were recorded in the rats treated with PHMB compared with the controls (*p*<0.2). However, histopathological examination revealed no lesions, suggesting that kidney function may not have been significantly compromised, even at the dose that caused 50% mortality (Greaves, 2007). A similar observation was made with PHMGH, a structurally-related compound (Asiedu-Gyekye *et al*., 2014).

In the subchronic toxicity study, three dose levels were tested and the results were devoid of clinical signs suggesting adverse events related to PHMB ingestion. Analysis of blood chemistry for red blood cells, mean corpuscular hemoglobin concentration and mean corpuscular hemoglobin levels did not show any differences between the PHMB doses tested and the controls. This shows that, at least for the period and at the dose levels tested, PHMB does not appear to exert adverse effects on the hematopoietic system. The surge in white blood cell count and neutrophils at the dose level of 8 mg/kg cannot be explained as the other dose levels tested yielded data comparable with the controls.

Biochemical analysis included AST, ALT, GGT and ALP as a reflection of liver function. The results suggest that the liver was in no serious toxic danger from the insult of the chemical. This was confirmed by the low level of degenerative lesions observed in the specimens at histopathological examination. In this study, the lipid profile in the PHMB-treated rats were similar to that of controls, suggesting that at the levels investigated, PHMB did not alter the metabolism of lipids. Other studies involving zebrafish have reported a rise in serum triacylglycerol level and fatty liver induction, which resulted in the death of the fish within 70 min when exposed to the working concentration of 0.3% PHMG (Kim *et al*., 2013).

Electrolyte imbalance mediates various pathological events (Hala *et al*., 2014: Jusmita *et al*., 2015). In this study, PHMB had no significant effect on electrolyte levels of the rats. This suggests that PHMB may not influence the electrolyte composition of the blood even when used subchronically. Mean urea values rather indicated that PHMB appeared to have a positive effect on renal function in the long run, as the urea levels appeared to be significantly lower than those of the controls. This observation was not entirely supported by the histopathological evaluation of kidney tissue, as some mild to severe degeneration lesions were observed in a few specimens. Although the level obtained for the group that produced the LD_50_ was high compared to the control, the difference was not statistically significant. It is rather interesting to note that creatinine levels were comparable between the treated and control groups (*p*<0.0009), which is in contrast to what was observed with PHMGH in rats (Asiedu-Gyekye *et al*., 2014). Urea levels, though, may be influenced by diet (Kang *et al*., 2015). This study has revealed that PHMB (LD_50_ 25.6 mg/kg bwt) may have a narrow margin of safety compared to PHMGH (LD_50_ 600 mg/kg bwt), which are both biocides and investigated for their possible use in treating water to make it potable.

In spite of these observations, the manufacturers recommend to use PHMB for water treatment at a dose of 8 mg/kg. Judging from the LD_50_ of both PHMB (25.6 mg/kg) and PHMGH (600 mg/kg) with their respective recommended doses from the manufacturer (Asiedu-Gyekye *et al*., 2014), the therapeutic indices of the two chemicals may stand at 3.2 and 50,000 respectively. On comparing the therapeutic indices, PHMGH appears to have a wider margin of safety than does PHMB. Yet it is likely that organ toxicity or cumulative toxicity may result after prolonged use more especially with PHMB and it might be safer to use PHMGH rather than PHMB in water treatment. A comparison of both the biochemical and the histopathological effects may give a reasonable idea about the possible adverse effects during long-term use of these biocides in humans.

## Conclusion

The median lethal concentration (LC_50_) of PHMB is 1.6 mL of 0.4%, which is equivalent to 6.4x10^3^ mg/L of 0.1% solution. Thus the LD_50_ is equivalent to 25.6 mg/kg. Subchronic toxicological studies showed few deleterious effects on the major organs examined, as seen from the results of the biochemical parameters evaluated. Gross pathology and histopathology also revealed that the integrity of the major organs was compromised especially at high doses compared with controls. This has implications for the use of PHMB in treating water to make it potable.

It is recommended that chronic toxicity studies be done to ascertain the long-term effect of PHMB.
